# Influence of the grain size of high explosives on the duration of a high conductivity zone at the detonation

**DOI:** 10.1038/s41598-019-48807-9

**Published:** 2019-08-22

**Authors:** Nataliya P. Satonkina

**Affiliations:** 1 Lavrentyev Institute of Hydrodynamics SB RAS, pr. ac. Lavrentyeva, 15, Novosibirsk, 630090 Russia; 2 Novosibirsk State University, Pirogova str., 1, Novosibirsk, 630090 Russia

**Keywords:** Chemical engineering, Chemical physics, Applied physics

## Abstract

At the detonation of condensed explosives, on the profile of electric conductivity is observed the area of high values, which is usually associated with the chemical reaction zone. The new interpretation of experimental data on the electrical conductivity allows one to diagnose the influence of the grain size on the charge structure and the reaction zone in the whole range of densities investigated. The reliability of the proposed hypotheses are investigated by the methods of statistical analysis. The level of confidence shows the consistency. The results of this paper are useful for the explosion physics, for the industrial production of nanodiamonds, for the miniaturization of explosive devices.

## Introduction

According to the Zel’dovich-von Neumann-Döring theory^1–3^, a detonation wave is a complex of the shock front, the adjacent chemical reaction zone (chemical peak, peak von Neumann), and the Taylor rarefaction wave which is separated from the chemical peak by the Chapman-Jouguet (CJ) point.

Determining the parameters of the chemical reaction zone at the detonation of condensed high explosives (HEs) is a complicated problem of the explosion physics which is important both from the theoretical and the practical point of view. Investigations of the reaction zone is complicated due to the aggressive environment and the high speed of the process: pressure of order of tens of GPa, temperature of several thousand degrees at the characteristic propagation velocity of the detonation of powerful HEs about 8 km/s. The high complexity of the investigation can be illustrated by the fact that the duration of the reaction zone for the same material measured measured by different methods and authors can differ by an order of magnitude^4–6^.

It is known that the charge preparation method, the charge structure, and the grain size influence significantly the reaction zone. The most prominent example is trinitrotoluene (TNT) for which the duration of the reaction zone for close densities but different preparation methods change from 0.33 *μ*s (cast charges with partial melting) to 0.19 *μ*s (pressed charges)^6^. A large amount of data is available on the critical diameter which, according to the Khariton’s principle^7^, is proportional to the duration of the reaction zone, and is also a characteristics of the chemical peak. In the work^8^, the critical diameters for the cast and pressed TNT of the same density of 1.6 g/cm^3^ differ by an order of magnitude (15–30 mm and 3–5 mm, correspondingly). Authors of the work^9^, where the influence of the grain size on the detonation at different densities was investigated, state that the size of a single grain determines the time of chemical reaction.

The works of Dremin *et al*.^6,10^ shows the opposite opinion about the absence of the influence of the grain size of TNT on the reaction zone. Thus, the data of different works contradict. In the work of Khasainov *et al*.^11^, the analytical dependences of the critical diameter on the grain size for a wide range of HEs constructed based on the data of works^12–14^. Also, the relations between the duration of the reaction zone, the critical diameter, and the shock-wave sensitivity were discussed. The last parameter is important first of all for practical purposes: the miniaturization of explosion devices expands the sphere of HE applications and increases the safety degree due to the decrease of the amount of HE necessary for the steady detonation. The prediction of shock-wave sensitivity for different grain size of HE is an actual research topic which can be emphasized by the increase of the number of related publications in recent years^15–19^. At present, the simulations of the transition from a shock-wave impact to the detonation are performed using the empirical kinetics. The knowledge of the characteristics of the reaction zone, in particular, its duration, will allow one to carry out simulation at the qualitatively new level.

In this work, the results are presented which are obtained using the alternative method for the diagnostics of the reaction zone by the electrical conductivity. Our previous investigations stated reliably the influence of the grain size for cyclotrimethylene-trinitramine (RDX), cyclotetramethylene-tetranitramine (HMX) and pentaerythritol tetranitrate (PETN) of a powder density which manifests itself by the shorter reaction zone^20^ and the significant decrease of the maximum mass velocity for smaller grains^21^. In the present work, the influence of the grain size on the time dependence of electrical conductivity was confirmed at the detonation of RDX in the whole range of densities investigated up to the maximum value.

## Experimental Data on the Electrical Conductivity at the Detonation of Condensed HEs

### Methodology

For the most of condensed HEs at the detonation, a region with high values is seen at the conductivity graph near the front^20,22–27^. This region is commonly related to the zone of chemical reaction^6,8,28–33^.

Experimental method to measure electrical conductivity at the detonation of condensed HEs and its foundation are given in work^22^ and in the Supplementary Materials.

**Figure 1:**
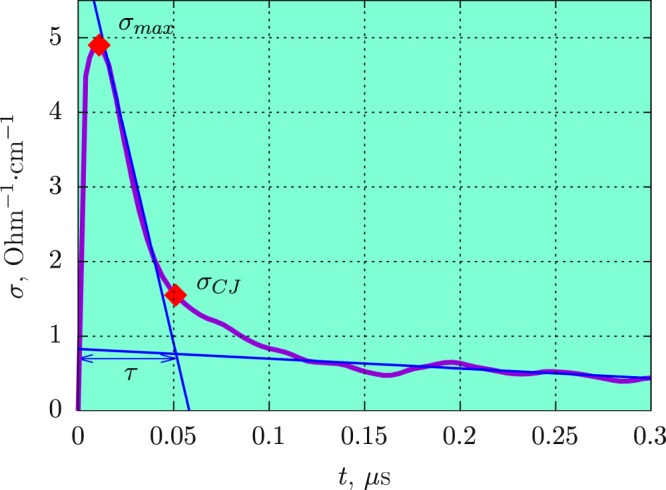
Conductivity profile at the detonation of cyclotrimethylene-trinitramine (RDX) with density *ρ* = 1.7 g/cm^3^. The determination procedure is shown of the duration of high conductivity zone *τ* and the conductivity at the CJ point *σ*_*CJ*_ and the maximum conductivity *σ*_*max*_.

A typical conductivity profile obtained at the detonation of cyclotrimethylene-trinitramine (RDX) is shown in Fig. 1: there is a fast increase to the maximum value *σ*_*max*_, then a decrease with a gradient dependent on the type of HE, and a slow change with a small value in the Taylor wave after the inflection point. The maximum conductivity *σ*_*max*_ corresponds to the region inside the chemical peak. We relate the inflection point *σ*_*CJ*_ at the conductivity profile *σ*(*t*) to the CJ point^34,35^. We define the duration *τ* of the high conductivity zone as the time between the beginning of the signal *σ*(*t*) and the moment of *σ*_*CJ*_ (the intersection of two approximation straight lines, Fig. 1).

All the data used in the analysis are presented in digital form in Supplementary Materials.

### On the interpretation of experimental data on the conductivity at detonation of powder density HEs

Earlier, we observed the influence of the grain size on the duration of the high conductivity zone at the detonation of RDX, HMX (cyclotetramethylene-tetranitramine) and PETN (pentaerythritol tetranitrate) of powder density. It was shown in work^20^ using the Student’s t-criterion that the probability of a determinative influence of the grain size on the duration is close to 100%. The value of the conductivity maximum *σ*_*max*_ was not taken into account at the analysis. Similar experiments of work^21^ with same HEs demonstrated shorter reaction zone at the mass velocity profile for smaller grain size.

**Figure 2:**
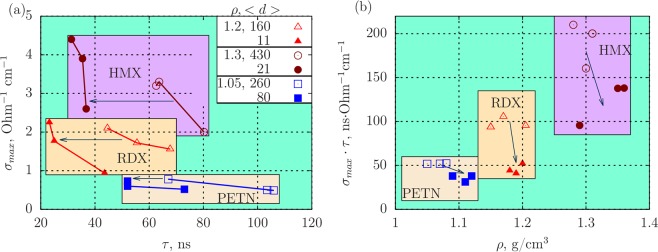
Influence of the grain size on the conductivity at the detonation of RDX, HMX and PETN of powder density: (**a)** maximum conductivity *σ*_*max*_ vs. the duration of the high conductivity zone *τ*, (**b**) function of the product of dependent quantities Φ = *σ*_*max*_ ⋅ *τ* vs. density of HE. Here, <*d*> is the average grain size in *μ*m, *ρ* is the density in g/cm^3^. Arrow shows the direction of the shift of experimental data for smaller grain size.

Figure 2a presents the experimental results in the coordinates *σ*_*max*_ vs. *τ*. The initial density and grain size of the HE are also shown. It can be clearly seen from the graph that the quantities *σ*_*max*_ and *τ* are dependent. The results for one HE with different grain sizes lie on distinct lines close to hyperbolas, which do not intersect in the range presented. For smaller grain size, the graph *σ*_*max*_(*τ*) is shifted to the left keeping close amplitude values *σ*_*max*_. This shift of *σ*_*max*_(*τ*) is the more pronounced the higher the ratio of standard grain size to the small one is. Thus, the analysis of the data presented in the paper of Ershov *et al*.^20^ using these coordinates shows that the data on electrical conductivity reveal the role of the grain size more clearly.

Since the dependence is hyperbolic, we can assume that the product Φ = *σ*_*max*_*τ* is constant for a fixed density. Figure 2b shows the density dependence of the function Φ. The data are grouped by the HE type, the initial grain size and the initial density and grain size. The largest scatter is observed for HMX with the maximum grain size which produces larger degree of inhomogeneity and leads to larger relative deviation of the value of Φ.

Thus, we can add to the results of work^20^ on the influence of the grain size on the duration of the high conductivity zone the following:

1. For smaller grain size, the maximum conductivity changes only slightly, where as the duration *τ* decreases significantly;
2. Values of *σ*_*max*_ and *τ* are dependent, their product has smaller statistical scatter, and it is determined by the density and the type of HE;
3. According to this interpretation, the influence of the grain size for three HEs manifests itself in the same way.

### Influence of the grain size on the conductivity at the detonation of RDX

The influence of the grain size at the powder density was especially looked for, but for lower porosity, the effect of the grain size was not expected. At the increase of density during the pressing process, the grains are crushed. This should change the structure of pores significantly comparing with the powder density. Thus, there was no reason to expect the influence of the grain size even at intermediate densities.

The effect was nevertheless discovered. As it was shown above, at the powder density, the interpretation of results using the variables *σ*_*max*_, *ρ* and *τ* leads to the decreasing statistical scatter and increases the sensitivity to the initial conditions.

**Figure 3:**
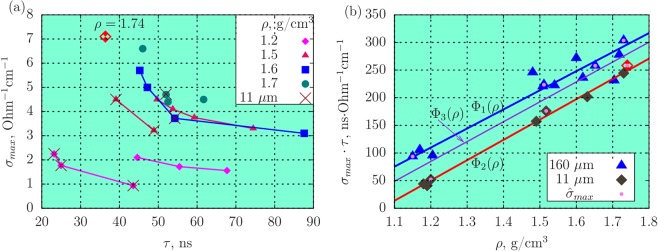
(**a)** Maximum value of conductivity *σ*_*max*_ vs. the duration *τ* of the high conductivity zone for RDX of different density, results for small grain size shown by ×; (**b)** product *σ*_*max*_*τ* vs. the density of RDX, symbol ${\hat{\sigma }}_{max}$ corresponds to the maximum value at a given density (leftmost points of the graphs (**a**)). Approximation functions: Φ_1_ is for the grain size 〈*d*〉 = 160 *μ*m, Φ_2_ – for 〈*d*〉 = 11 *μ*m, Φ_3_ is the approximation for all grain sizes.

Among all the HEs investigated, most experimental data are obtained for RDX. Figure 3a shows all the experimental results on the conductivity for RDX^20,22–24^ in the coordinates *σ*_*max*_ vs. *τ* for the range of density *ρ* ± 0.3 g/cm^3^. Most of these data are obtained for the grain size 160 *μ*m, results for 11 *μ*m are marked out. Same as for low-density HEs, the hyperbolic dependence *σ*_*max*_(*τ*) is clearly seen for close values of density. Figure 3b shows the product Φ(*ρ*) = *σ*_*max*_*τ* vs. the real density. For each point in the graph 3a, there is a corresponding point in 3b.

The meaning of the product *σ*_*max*_ ⋅ *τ* is an estimate proportional to the conduction *G* in the region of high conductivity. The conduction is related to the resistance *R* as *G* = 1/*R*. From the experimental point of view, the hyperbolic dependence *σ*_*max*_(*τ*) can be explained by the presence of inhomogeneities in the charge structure, which are negligible only for a single crystal.

The pronounced effect of the grain size is observed in Fig. 3a not only for low-density RDX, but also for the density *ρ* = 1.5 g/cm^3^: the dependence *σ*_*max*_(*τ*) is shifted towards the y-axis with the same amplitude values of conductivity. However, it is not possible to single out experiments with small grains for the density *ρ* > 1.5 g/cm^3^ in Fig. 3a. In Fig. 3b, the same results presented in different coordinates are separated according to the grain size.

Linear density dependences of Φ_1_(*ρ*) for the average grain size 〈*d*〉 = 160 *μ*m and Φ_2_(*ρ*) for small grains are significantly separated, the function Φ_2_(*ρ*) lies lower than Φ_1_(*ρ*). Considering the slight influence of the grain size on the value of *σ*_*max*_ for a fixed density, the shift of Φ can be interpreted as the decrease of the high conductivity zone for the whole range of densities. Since *τ* is related to the reaction zone, the shift of Φ for smaller grain size means the increase of the detonation ability^7^, which agrees with the work^36^. Small scatter of the values of Φ_2_ for small grain size shows the role of hot spots the concentration of which is in this case significantly higher.

The points corresponding to the maximum electrical conductivity ${\hat{\sigma }}_{max}$ (upper left values at the graphs for each *ρ* in 3a) are marked out in the graph 3b. The values of Φ_1_ corresponding to these points have lower scatter relative to the overall linear dependence than other data for the common grain size.

The point in Fig. 3a with the coordinate (*τ* = 36 ns, *σ*_*max*_ = 7.1 Ohm^−1^cm^−1^) with density *ρ* = 1.74 g/cm^3^ deserves special discussion. When HE is pressed into the case for experiments, the plunger rests on the limiting rings thus providing the prescribed density. In this experiment, however, the plunger rested on the HE leading to the obtaining the maximum achievable at the pressing of a pure HE density. We can assume that the decrease of the duration to 36 ns is related to the maximum achievable crushing of HE particles due to the action of a high pressure and the similarity of the pore structure to charges with fine grains.

This point at the graph Fig. 3b belongs to Φ_2_(*ρ*) for small grains. There was a single such experiment, and this assumption was not tested.

Thus, considering the duration of the high conductivity zone *τ* and the maximum value *σ*_*max*_ leads to the increasing error. In order to obtain the maximum value of *σ*_*max*_ for a given density, it is necessary to find the maximum value of several experiments for the same density, which can be treated as the result of the minimum distortion and the best homogeneity for a given density. The duration *τ* corresponding to this value determines the chemical reaction time. It should be noted that in this interpretation, the minimum duration is an upper estimate of the real value which can be obtained by increasing the statistics.

Since the value of *σ*_*max*_ depends on the grain size only slightly, we can assume that it is determined by the density (the amount of the reacting HE) while the duration *τ* of the high conductivity zone contains information on the HE grain size.

In this interpretation, the electrical properties are sensitive to such subtle phenomena as a change of the characteristics of inhomogeneities for the same density. The role of the grain size can be revealed by considering the data in the coordinates *σ*_*max*_*τ* vs. *ρ*. The separate use of the duration of the high conductivity zone and the maximum value *σ*_*max*_ leads to the loss of information on the influence of the grain size. It was shown that the maximum value ${\hat{\sigma }}_{max}$ from several experiments can be considered as the most reliable one. The integral value of Φ(*ρ*) is subject to a smaller statistical scatter.

## Analysis of Experimental Data

Since some of the assumption of the present work were not discussed earlier neither in the experimental papers, nor theoretically, we need to make a more detailed overview of the proposed hypotheses going into particularities of the statistical analysis.

Let’s test the hypotheses proposed using the most universal and sensitive *χ*^2^ criterion^37^. The results are shown in Table 1 (the data used for this analysis are contained in the Supplementary Materials). The following notation is assumed: *a* and *b* are the fitting parameters for a function of the form *aρ* + *b*; in the column *χ*^2^/*N*_*dof*_, the numerator is the number of events, the denominator – the number of parameters; *N*_*dof*_ is the number of degrees of freedom, *P*(*χ*^2^) is the probability of the validity of the hypothesis; the absolute instrumental error is *ε* = 12%, Φ_1−3_ are the approximation functions for the product *σ*_*max*_*τ* vs. *ρ*.

**Table 1:** Fitting results for the function Φ(*ρ*) = *σ*_*max*_*τ* for the density dependence of the type *aρ* + *b*.

N	hypothesis	*a*	*b*	*χ*^2^/*N*_*dof*_	*P*(*χ*^2^), %	notes
1	Φ_1_(*ρ*)	345.39	−304.68	8.95/13-2	62.7	〈*d*〉 = 160 *μ*m
2	Φ_2_(*ρ*)	367.41	−390.58	1.75/7-2	88.2	〈*d*〉 = 11 *μ*m
3	Φ_3_(*ρ*)	359.76	−347.03	164.3/21-2	4 ⋅ 10^−23^	all points

The function Φ_1_(*ρ*) is the approximation over all the points with the common grain size, Φ_2_(*ρ*) is the approximation for small grains, and Φ_3_(*ρ*) is the approximation over all the points regardless the grain size. For the larger number of events regardless the grain size, the significance level for the null hypothesis (the product *σ*_*max*_*τ* does not depend on the grain size) is the value close to zero (Table 1, line 3). This probability increases sharply and tends to 100%, if the data are grouped according to the chosen principle of the influence of the grain size (functions Φ_1_(*ρ*) and Φ_2_(*ρ*)).

We can find the probability for the value of *σ*_*max*_*τ*(*ρ*) to belong to the single dependence Φ_3_ regardless the grain size using the Wilkokson criterion^38^. The number of events for the sampling is 7 for small grains and 14 for the common grain size. The number of inversions is 5 and 93, correspondingly. The probability of the validity of the hypothesis that these two samplings belong to a single sequence is lower than 0.2%.

Thus, the points of the graph Fig. 3b are separated according to the grain size, and they belong to different dependencies with a high degree of reliability.

## Discussion

**Figure 4:**
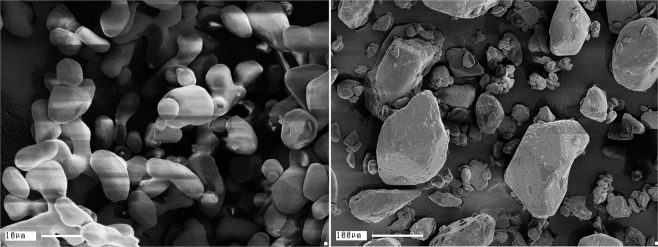
Photographs of RDX grains. Left is the fine-grained powder with the average grain size 〈*d*〉 = 11 *μ*m, right is the common powder with the grain size 〈*d*〉 = 160 *μ*m.

Photographs of the RDX grains of different size used in the experiments are shown in Fig. 4. Particles with the average size 〈*d*〉 = 11 *μ*m mostly have a rounded shape, and the grains have close sizes. This provides a high degree of homogeneity of a charge. The common powder also contains small particles, but the main part of HE is concentrated in large ones, which determine the duration of the chemical reaction zone.

It is commonly assumed that particles are crushed during the pressing, and the influence of the grain size can be neglected already for an intermediate density. Our data shows that the information on the initial grain size of HE is preserved after the pressing up to the maximum achievable density. The influence of the grain size on the electrical conductivity at the detonation of RDX is revealed through the integral quantity Φ(*ρ*) = *σ*_*max*_*τ*. For smaller grains, the shift of Φ(*ρ*) occurs, which corresponds to the decrease of the duration of the high conductivity zone. The inclination angle of the linear dependence remains the same (Fig. 3). The probability that the point for small grain size belong to the linear dependence Φ_2_(*ρ*) is nearly 90% (Table 1).

Experimental testing of the change of the structure and the grain size at the pressing should be performed using methods of bulk diagnostics, i.e., the tomography. Only the surface is available at the microscopic investigation, where the grains occupy distinguished positions, and the impact of the pressing surface is not identical to the inner stress and to the interaction of grains at the compactification.

In order to support our result by the data of literature, let’s remind the Khariton’s principle^7^, which states that the duration of a chemical reaction zone is proportional to the critical diameter.

Table 2 presents the data on the influence of the HE grain size on the critical diameter and the duration of the high conductivity zone for the powder density. The following notation is assumed: *ρ*, *ρ*_*σ*_ is the density, $\langle {d}_{1,2}^{\text{'}}\rangle$, 〈*d*_1,2_〉 is the average grain size, 1 corresponds to larger grains, ${d}_{cr}({d}_{1,2}^{\text{'}})$ is the critical diameter of a cylindrical charge for the grain size given in brackets^12,13^, *τ*(*d*_1,2_) is the minimum for a given density duration of the high conductivity zone for the grain size given in brackets^20^. Let’s remind that the minimum of *τ* correspond to the maximum value of *σ*_*max*_ and to the best homogeneity. A prominent compliance is observed by the comparison of the ratio of critical diameters and the duration of the high conductivity zone. Thus, the correctness of the interpretation of the shift of *σ*_*max*_*τ* for smaller grains at the powder HE density is confirmed by the data of works^12,13,20,21^.

**Table 2:** Influence of the grain size on the critical diameter (data of works^12,13^) and the duration of the high conductivity zone (data of work^20^) for HEs of a powder density.

HE	*ρ*, g/cm^3^	$\langle {{{\boldsymbol{d}}}^{^{\prime} }}_{{\boldsymbol{1}}}\rangle$, *μ*m	$\langle {{{\boldsymbol{d}}}^{^{\prime} }}_{{\boldsymbol{2}}}\rangle$, *μ*m	${{\boldsymbol{d}}}_{{\boldsymbol{c}}{\boldsymbol{r}}}({{{\boldsymbol{d}}}^{^{\prime} }}_{{\boldsymbol{1}}})/{{\boldsymbol{d}}}_{{\boldsymbol{c}}{\boldsymbol{r}}}({{\boldsymbol{d}}}_{{\boldsymbol{2}}}^{^{\prime} })$, [*d*_*cr*_] = mm	ref.	*ρ*_*σ*_, g/cm^3^	〈*d*_1_〉, *μ*m	〈*d*_2_〉, *μ*m	*τ*(*d*_1_)/*τ*(*d*2), [*τ*] = ns
PETN	1.0	240	100	3.5/1.5 = 2.3	^12^	1.1	260	80	67/52 = 1.3
RDX	1.0	180	80	4.4/2.4 = 1.8	^12^	1.2	160	11	45/23 = 2.0
HMX	1.0	530	52	8.0/4.0 = 2.0	^13^	1.3	430	21	64/31 = 2.1

The slight effect of the decrease of the duration *τ* is observed for PETN. This can be related to the large grain size 〈*d*_2_〉 = 80 *μ*m, to the high degree of inhomogeneity, to the different behaviour of PETN for changing grain size comparing to other HEs obtained in work^36^, and to the poor statistics. Nevertheless, the influence of the grain size on the duration of the high conductivity zone is registered for PETN with a high degree of reliability (Fig. 2).

The influence of the grain size for high densities was obtained in work^36^, where in a planar case, the decrease of the grain size leaded to the increase of the receptivity to a detonation and the decrease of the critical layer thickness (the analogue of the critical diameter in a planar case). Results of this work are summarized in Table 3, the notation *h*_*cr*_ corresponds to the critical HE layer thickness, the ratio close to 2.5 is higher than the one listed Table 2, which can be explained by the geometry. The effect of the grain size is even more pronounced in the planar case.

**Table 3:** Data on the critical layer width for high-density HEs^36^ at the grain size of $\langle {d^{\prime\prime} }_{1}\rangle =100$ *μ*m and $\langle {d^{\prime\prime} }_{2}\rangle =3.5$ *μ*m.

HE	*ρ*, g/cm^3^	${{\boldsymbol{h}}}_{{\boldsymbol{c}}{\boldsymbol{r}}}({{\boldsymbol{d}}^{\prime\prime} }_{{\boldsymbol{1}}})/{{\boldsymbol{h}}}_{{\boldsymbol{c}}{\boldsymbol{r}}}({{\boldsymbol{d}}^{\prime\prime} }_{{\boldsymbol{2}}})$, [*h*_*cr*_] = mm
PETN	≈1.63	0.22/0.10 = 2.2
RDX	≈1.68	0.45/0.18 = 2.5
HMX	≈1.81	0.81/0.32 = 2.5

The decrease of the reaction zone duration and the increase of the reaction rate for smaller initial grain size was obtained numerically in the work^39^. In the work^11^, the analytical relation was obtained between the critical diameter and the grain size through the surface area in the whole density range. The necessity of an experimental verification is mentioned there, and the results our work can be considered as such verification. Since the kinetics of the reaction zone governs the condensation of carbon at the detonation^40–42^, the influence of the grain size of HE on the size of detonation nanodiamonds is also an evidence of the intensification of the chemical reaction for smaller grain size^43,44^.

The total data considered, which were obtained by different methods, are in a good agreement. The relatively simple method of electrical conductivity with the high sensitivity and the modest statistics allows us to diagnose experimentally the decrease of the reaction zone duration for smaller grain size. The dependence Φ(*ρ*) is determined just by the grain size, and it shows high repeatability and minimum scatter due to the increase of homogeneity. The use of a fine-grained HE for making even high density charges by pressing leads to the shorter reaction zone and the higher homogeneity of the charge structure.

The results of this paper are useful first of all for the explosion physics. The can be used in the investigation of the kinetics of the reaction zone in powerful HEs and for the development of the theory of hot spots. The results are actual for the industrial production of nanodiamonds especially for the development of a technology of the manufacture of a product with the desired properties. The results are also useful for the miniaturization of explosive devices and the increase of safety at the handling of HEs.

## Conclusion

The proposed in this work interpretation of the experimental data on the distribution of electrical conductivity at the detonation of condensed HEs is analyzed using statistical criteria. The degree of reliability supports the consistency of the hypothesis on the influence of the grain size of HE on the structure of charge and the chemical reaction zone in the whole range of densities investigated. Results of the work agree well with both the data of numerical simulations and the experimental results.

The author is grateful to A.P. Ershov for many years of a fruitful collaboration, to K.Yu. Todyshev and E.R. Pruuel for important discussion and constructive criticism, to D.A. Medvedev for the help in the preparation of the paper.

## Supplementary information

Dataset 1

## Data Availability

The data that support the plots within this paper and other findings of this study are available in additional material and from the corresponding author upon reasonable request.
