# Supplementary material for: Influence of the grain size of high explosives on the duration of a high conductivity zone at the detonation
Source: Sci Rep. 2019 Aug 22;9:12256. doi: 10.1038/s41598-019-48807-9 (PMC6706435; doi:10.1038/s41598-019-48807-9)
Supplement: Supplementary file 1 — Dataset 1 [file 41598_2019_48807_MOESM1_ESM.pdf]

Additional materials to the article Influence of the grain size of high explosives on the duration of a high conductivity zone at the detonation

Nataliya P Satonkina [1,2,*]

Lavrentyev Institute of Hydrodynamics SB RAS, Novosibirsk, 630090, Russia

[2] Novosibirsk State University, Novosibirsk 630090, Russia

September 4, 2018

Experimental setup

We employed a coaxial arrangement of electrodes (Fig. 1). A charge with a diameter of $b = 8$ mm was pressed into a thick-wall copper confinement (*1*, *2*) 40 mm in diameter. Axial copper electrode *3* with a diameter of $c = 2$ mm was secured in Plexiglas stopper *4* fixed with hollow bolt *5*. A cavity in the external electrode accommodated electroconductivity sensor 7, a toroidal coil. The coil–cavity contour mutual inductance M was 15 nH. Parts *1* and *2* of the external electrode were attached to each other via a screw joint. The thickness of the slit between parts *1* and *2* was preset by dielectric layer *6* (0.3 mm of Teflon or 0.3–1.0 mm of Plexiglas). When the detonation wave arrives at the coaxial electrode, the cell circuit becomes connected by the conducting region behind the detonation front. The electric current passing through the cell flows around the sensor cavity, thereby inducing a magnetic flux through the coil and, hence, a voltage pulse across its terminals. Later, when the detonation front travels past the slit, part of the current begins to flow via external electrode *2*. As a result, the strength of the current overflowing the cavity and, consequently, magnetic flux decrease,

*Corresponding author. Tel: +7 9137400572. E-mail: snp@hydro.nsc.ru

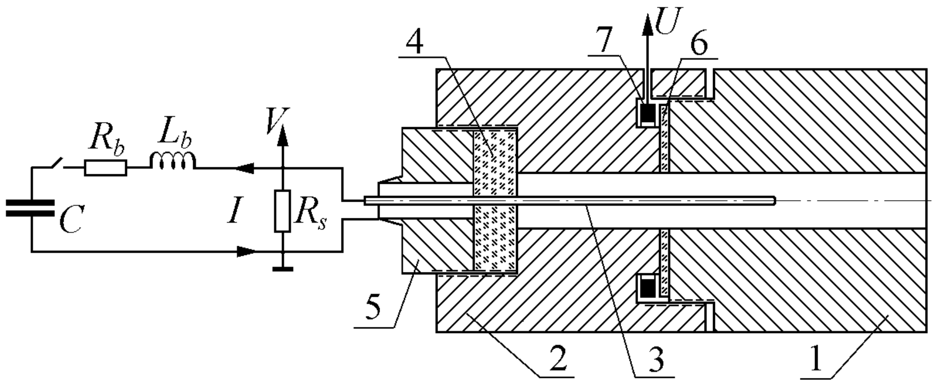

Figure 1: Experimental setup.

producing in the sensor a voltage pulse U of opposite polarity, with the electric conductivity in the plane of the slit being proportional to $U(t)$ [1]:

$$\sigma(x) = \frac{\ln(b/c)}{2\pi DM}\frac{U(t)}{V}$$

Here, $x = Dt$ is the distance the front moved off from the slit by time t after passing it, D is the detonation velocity, V is the voltage across the electrodes produced by the feeding current I passing through resistor R_s connected in parallel with the conducting region of the charge. The cell was fed by capacitor C (100 μF, 1000 V) connected to the circuit through a chain composed of resistor R_b and induction coil L_b.

The actual voltage U differs somewhat from the experimentally measured one U in because of the effect of coil self-inductance ($L \approx 1$ μH), being related to it by the formula $U = U_{in} + (L/R)dU_{in}/dt$, where R is the input resistance of the oscilloscope (50 Ω). Since the calculation procedure involved differentiation, the signal was spline-smoothed to suppress small-scale noises.

The illustration of the registration process is contained in the file mov.gif.

The cell depicted in Fig. 1 renders electric current spreading unimportant, since the conductance of a strictly specified layer (between the detonation wave front and the plane of the slit) is measured. Since the electric field vector is perpendicular to the electroconductivity gradient nearly everywhere, the space charge effect is negligible. Another advantage of the cell is its ability to keep the intensity of gasdynamic perturbations as low as possible. A thin slit causes only a slight rarefaction, in contrast to shock waves typical of most experimental setups.

The resolution of the method is determined by the slit width $2a$. Analytical estimates and direct numerical calculations of the electric current distribution over

the cell space (appendix) demonstrated that an optimal estimate of the experimental resolution is one-quarter of the slit width, $a/2 = 0.075$ mm ($2a = 0.3$ mm).

The differential scheme used in [1] included an external measuring contour, a short wire with an inductance of 50 nH. The main advantage of the new, completely coaxial scheme is a fairly low inductance of the cavity contour L_c (less than 1 nH).

The charges were initiated by electric detonators through Plexiglas stopper *8* (Fig. 1) with a 2.4-mm axial channel filled with RDX. Thus, the main charge was initiated 40 mm from the plane of the slit and within 1.2 mm from the axis. This deviation might cause a scatter in the distance of travel of the wave to the slit less than 0.24 mm (if the detonation front is a segment of a sphere with the center at the point of initiation). In reality, the effect of the walls makes this scatter smaller—according to electroconductivity measurements, the scatter in pulse widths was less than 0.05 mm.

More detailed information on the scheme can be found in work [2].

References

References

[1] A. P. Ershov, P. I. Zubkov, and L. A. Luk'yanchikov, Measurements of the electrical conductivity profile in the detonation front of solid explosives Combustion, Explosion, and Shock Waves. 1974. V. 10(6). P. 776-782.

[2] A. P. Ershov, N. P. Satonkina, G. M. Ivanov. Electroconductivity Profiles in Dense High Explosives Russ. J. Phys. Chem. B. 2007. V. 26(12). P. 21.

Experimental data of electric conductivity at detonation of the cyclotrimethylene-trinitramine (RDX), cyclotetramethylene-tetranitramine (HMX), pentaerythritol tetranitrate (PETN)

In the Table 1, the data for RDX, HMX and PETN are presented. The notation is following: $\langle d \rangle$ – average grain size, ρ – initial density, D – detonation velosity, τ

– duration of high electrical conductivity zone, σ_{CJ} and σ_{max} is the conductivity in the CJ point and the maximum, correspondingly.

The other data that support the findings of this study are available from the corresponding author upon reasonable request.

Table 1: The data for RDX, HMX and PETN.

explosive	$\langle d \rangle$, μm	shot #	ρ, g/cm^3	D, km/s	σ_{max}	σ_{CJ}	τ, ns
RDX	160	71	1.54	7.76	3.75	1.4	59
		72	1.65	8.18	5.7	1.8	45
		73	1.73	8.48	6.6	1.5	46
		74	1.74	8.52	7.1	2.0	36
		82	1.6	7.99	3.1	1.0	88
		83	1.72	8.43	4.5	1.5	62
		84	1.48	7.52	3.3	1.0	74
		91	1.62	8.05	5	1.5	47
		92	1.7	8.38	4.4	1.5	52
		102	1.20	6.49	1.72	0.3	55
		105	1.17	6.35	1.56	0.4	68
		130	1.51	7.65	4.5	1.1	50
		135	1.51	7.63	4.1	1.0	54
		143	1.15	6.28	2.1	0.4	45
	11	97	1.63	8.11	3.72	1.4	54
		98	1.49	7.57	3.22	1.4	49
		99	1.73	8.46	4.7	1.6	52
		132	1.52	7.67	4.5	1	39
		140	1.19	6.43	0.94	0.2	44
		141	1.18	6.41	1.77	0.3	25
		144	1.2	6.47	2.25	0.5	23
HMX	430	146	1.28	6.75	3.3	0.5	64
		148	1.30	6.86	2.0	0.7	80
		150	1.31	6.87	3.2	0.9	63
	21	147	1.29	6.80	2.6	0.7	37
		149	1.36	7.06	3.9	1.0	35
		151	1.35	7.03	4.4	1.0	31
PETN	260	152	1.05	5.70	0.50	0.15	102
		154	1.07	5.76	0.49	0.12	106
		156	1.08	5.82	0.78	0.18	67
	80	153	1.11	5.92	0.60	0.18	52
		155	1.09	5.85	0.52	0.10	73
		157	1.12	5.96	0.73	0.20	52
